# Pediatric Emergency Visits and Short-Term Changes in PM_2.5_ Concentrations in the U.S. State of Georgia

**DOI:** 10.1289/ehp.1509856

**Published:** 2015-10-09

**Authors:** Matthew J. Strickland, Hua Hao, Xuefei Hu, Howard H. Chang, Lyndsey A. Darrow, Yang Liu

**Affiliations:** 1 Department of Environmental Health,; 2 Department of Biostatistics and Bioinformatics, and; 3 Department of Epidemiology, Rollins School of Public Health, Emory University, Atlanta, Georgia, USA

## Abstract

**Background:**

Associations between pediatric emergency department (ED) visits and ambient concentrations of particulate matter ≤ 2.5 μm in diameter (PM2.5) have been reported in previous studies, although few were performed in nonmetropolitan areas.

**Objective:**

We estimated associations between daily PM2.5 concentrations, using a two-stage model that included land use parameters and satellite aerosol optical depth measurements at 1-km resolution, and ED visits for six pediatric conditions in the U.S. state of Georgia by urbanicity classification.

**Methods:**

We obtained pediatric ED visits geocoded to residential ZIP codes for visits with nonmissing PM2.5 estimates and admission dates during 1 January 2002–30 June 2010 for 2- to 18-year-olds for asthma or wheeze (n = 189,816), and for 0- to 18-year-olds for bronchitis (n = 76,243), chronic sinusitis (n = 15,745), otitis media (n = 237,833), pneumonia (n = 52,946), and upper respiratory infections (n = 414,556). Daily ZIP code–level estimates of 24-hr average PM2.5 were calculated by averaging concentrations within ZIP code boundaries. We used time-stratified case-crossover models stratified on ZIP code, year, and month to estimate odds ratios (ORs) between ED visits and same-day and previous-day PM2.5 concentrations at the ZIP code level, and we investigated effect modification by county-level urbanicity.

**Results:**

A 10-μg/m3 increase in same-day PM2.5 concentrations was associated with ED visits for asthma or wheeze (OR = 1.013; 95% CI: 1.003, 1.023) and upper respiratory infections (OR = 1.015; 95% CI: 1.008, 1.022); associations with previous-day PM2.5 concentrations were lower. Differences in the association estimates across levels of urbanicity were not statistically significant.

**Conclusion:**

Pediatric ED visits for asthma or wheeze and for upper respiratory infections were associated with PM2.5 concentrations in Georgia.

**Citation:**

Strickland MJ, Hao H, Hu X, Chang HH, Darrow LA, Liu Y. 2016. Pediatric emergency visits and short-term changes in PM2.5 concentrations in the U.S. state of Georgia. Environ Health Perspect 124:690–696; http://dx.doi.org/10.1289/ehp.1509856

## Introduction

Efforts to model ambient air quality have progressed rapidly in recent years. One area that has been advanced is the estimation of particulate matter ≤ 2.5 μm in diameter (PM_2.5_) concentrations from satellite remote sensing and land use parameters. The MAIAC (Multi-Angle Implementation of Atmospheric Correction) data product derived from MODIS (Moderate Resolution Imaging Spectroradiometer) aerosol optical depth measurements allows for estimation of PM_2.5_ at 1-km^2^ spatial resolution (Lyapustin et al. 2011a, 2011b, 2012). This fine spatial scale enables identification of local pollution gradients that are poorly characterized at coarser resolutions, such as gradients near freeways.

Another advantage of air quality models that incorporate satellite data is the ability to estimate pollutant concentrations in locations where monitoring data are sparse or nonexistent. The composition of PM_2.5_ differs between urban and rural locations (U.S. EPA 2009), and although some epidemiologic studies have been conducted in rural areas (Noonan et al. 2012; Rappold et al. 2011; Weir et al. 2013), overall there is a lack of information about rural PM_2.5_ health effects. Further research is needed to investigate the extent to which associations with rural PM_2.5_ differ from urban PM_2.5_ associations.

Epidemiologic studies based on PM_2.5_ estimates from satellite data appear in the literature, and associations have been reported with diverse outcomes such as mortality, cardiorespiratory hospitalizations, myocardial infarctions, and pregnancy outcomes (Kloog et al. 2012a, 2012b, 2013; Madrigano et al. 2013). In our study we investigate associations between short-term changes in ambient PM_2.5_ concentrations estimated from MAIAC remote sensing and pediatric emergency department (ED) visits for asthma, bronchitis, chronic sinusitis, otitis media, pneumonia, and upper respiratory infections in the U.S. state of Georgia. To investigate whether PM_2.5_ health associations differ by urbanicity, we categorized ZIP codes according to county-level urbanicity, and estimated pollutant associations separately for three levels of urbanicity.

## Material and Methods

### Health Data

Individual-level data on pediatric ED visits in Georgia during 1 January 2002 through 30 June 2010 were obtained from the Georgia Hospital Association (*n* = 8,252,559 ED visits from 150 hospitals). Outcomes were defined using *International Classification of Diseases, 9th Revision* (ICD-9) codes. Case definitions were asthma or wheeze among children age 2–18 years (ICD-9 code 493 or 786.07 in any diagnosis field), bronchitis (age 0–18 years) (primary ICD-9 code 466.0 or 490), chronic sinusitis (age 0–18 years) (primary ICD-9 code 473), otitis media (age 0–18 years) (primary ICD-9 code 381 or 382), pneumonia (age 0–18 years) (primary ICD-9 code 480–486), and upper respiratory tract infection (age 0–18 years) (primary ICD-9 code 460–465 or 477) and absence of ICD-9 codes for asthma or wheeze (ICD-9 code 493 or 786.07) in the other diagnosis fields. Our *a priori* case definition for asthma or wheeze uses all ICD-9 diagnosis fields because there is between-hospital variability in primary diagnosis coding practices for children who present with both asthma and a respiratory infection, and we chose to classify these ED visits as asthma. These visits were excluded from the upper respiratory infection case definition. Records contained information on the date of the ED visit and the ZIP code of the patient’s home address. County-level urbanicity was assigned using the U.S. National Center for Health Statistics Classification Scheme (Ingram and Franco 2012). In this approach, each county is first classified as metropolitan or nonmetropolitan using the Office of Management and Budget’s 2005 list of metropolitan statistical areas (Office of Management and Budget 2005). In our analysis, we examined three categories of urbanicity, according to the characteristics of the county into which each ZIP code falls: “large metropolitan” (metropolitan counties with > 1 million residents) (*n* = 207 ZIP codes), “medium or small metropolitan” (metropolitan counties with 250,000–999,999 residents or < 250,000 residents, respectively) (*n* = 175 ZIP codes), and “nonmetropolitan” (counties not in a metropolitan statistical area) (*n* = 309 ZIP codes).

### Air Quality and Meteorological Data

MAIAC aerosol optical depth (AOD) values during the study period from the Aqua MODIS (overpasses at ~ 1330 hours local time) and Terra MODIS (overpasses at ~ 1030 hours local time) instruments were first combined daily to improve spatial coverage using a linear regression approach (Hu et al. 2014a; Puttaswamy et al. 2014). To predict daily PM_2.5_ concentrations, we developed a two-stage spatiotemporal model. The first stage is a linear mixed-effects model with day-specific random intercepts and slopes for AOD and meteorological fields to account for the temporally varying relationship between observed PM_2.5_ and AOD. The second stage is a geographically weighted regression model fitted monthly that can generate a continuous surface of PM_2.5_ estimates for all the grid cells with AOD retrievals. This model was fitted annually, and we obtained model-fitting *R* ^2^ ranging from 0.71 to 0.85 and daily mean prediction error of 1.73 to 2.50 μg/m^3^. Details of model structure and performance evaluation are provided elsewhere (Hu et al. 2014b). Daily ZIP code–level PM_2.5_ concentrations were calculated by averaging PM_2.5_ concentrations from all 1-km grid cells that were completely contained within each ZIP code boundary. Estimates of mean daily near-surface air temperature and near-surface humidity at 1/8th-degree resolution (~ 13-km grids) were obtained from the National Land Data Assimilation System website (http://ldas.gsfc.nasa.gov/nldas/) (Cosgrove et al. 2003). ZIP codes were linked to the meteorological fields based on ZIP code centroid.

### Statistical Analyses

Time-stratified case-crossover models (Janes et al. 2005), with stratification by ZIP code, year, and month, were used to estimate odds ratios (OR) between ZIP code–level daily counts of ED visits and daily ZIP code level estimates of PM_2.5_ concentrations. Models included cubic polynomials for same day (lag 0) mean temperature, lag 0 mean humidity, and day of year (1,…,366); indicators for day of week, warm season (May–October vs. November–April), holiday, and lag holiday (indicating whether 1 of the previous 2 days was a holiday); and product terms between the warm season indicator and the temperature cubic polynomial, humidity cubic polynomial, day of week indicators, holiday indicators, and lag holiday indicators to allow for seasonal interactions in the effects of these confounders. Although the motivation for stratification in case-crossover models is to control for confounding by season and trend, some outcomes (e.g., asthma) have marked within-month trends in incidence (Johnston et al. 2006); residual confounding could occur if these months also exhibit trends in PM_2.5_ concentrations. The smooth day-of-year function is included to control for this potential confounding. ORs corresponding to a 10-μg/m^3^ increase in lag 0 PM_2.5_ concentrations were assumed to be linear on the logit scale. In separate models we also estimated ORs for a 10-μg/m^3^ increase in lag 1 PM_2.5_ concentrations. Associations with lag 0 PM_2.5_ were estimated for Georgia overall and for each of the three levels of urbanicity.

AOD-based estimation of PM_2.5_ offers high spatial granularity; however, missing data are frequent due to cloud cover and errors in AOD retrieval. Therefore, we began by evaluating the influence of the extent of missing PM_2.5_ data on our exposure estimates and on our estimates of association. To evaluate the influence of missing data on exposure estimates, we limited our data set to ZIP codes that contained a PM_2.5_ monitoring station. For each ZIP code we compared the measured 24-hr average PM_2.5_ concentration at the monitor with the average of all nonmissing 1-km AOD-based PM_2.5_ estimates for that ZIP code. We also compared the monitor measurement with the single AOD-based PM_2.5_ estimate for the single grid that coincided with the location of the monitor. To display these results, each within–ZIP code daily comparison was binned according to the proportion of nonmissing 1-km AOD-based PM_2.5_ estimates for that ZIP code. To evaluate the influence of missing data on associations between PM_2.5_ and the outcomes, we used the data set of all ZIP codes. We compared associations estimated using only data from ZIP codes with complete 1-km grid PM_2.5_ estimates for each ED visit and corresponding reference days (i.e., 0% missing); using data from ZIP codes with < 10%, < 20%,…,< 90% of grid estimates with missing data; and using data from any ZIP code with at least one 1-km grid PM_2.5_ estimate on each ED visit day and corresponding reference days (i.e., < 100% missing).

We also conducted sensitivity analyses to examine the impact of missing data on OR estimates. In the two most extreme cases, we either *a*) only analyzed ZIP codes that had complete 1-km grid PM_2.5_ estimates or *b*) analyzed as many data as possible (i.e., so long as a ZIP code had at least one 1-km grid PM_2.5_ estimate it was included in the analysis). We also examined results for a range of missing data scenarios between these two extremes. These sensitivity analyses informed our decision to exclude a ZIP code when > 70% of the grid estimates were missing on a given day.

## Results

**Figure 1:**
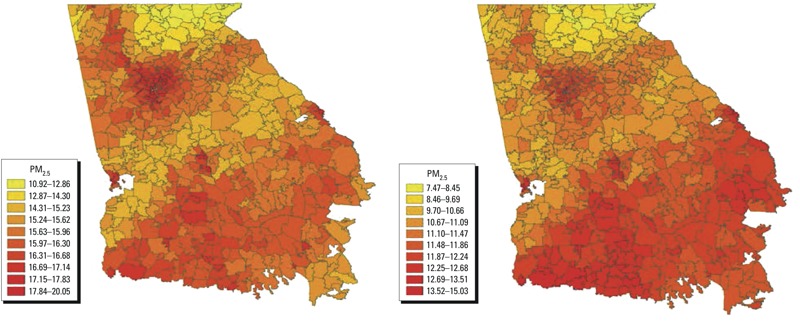
ZIP code–level mean PM_2.5_ concentrations during 1 January 2002 through 30 June 2010 during May–October (left panel) and November–April (right panel).

Mean PM_2.5_ concentrations by season, averaged across the study period, for the 691 Georgia ZIP codes included in analyses are shown in Figure 1. Concentrations tended to be highest in urban Atlanta ZIP codes (in the north-central part of the state) and in southern Georgia, where prescribed fires as well as agricultural emissions, such as ammonium from fertilizer use, are more common. Also conveyed in Figure 1 is the heterogeneity in ZIP code sizes throughout Georgia. Approximately 34% of ZIP codes contain between 1 and 49 1-km grid cells, 32% contain 50–149 cells, 20% contain 150–299 cells, and 14% contain 300–1,224 cells. Distributions of daily ZIP code–level PM_2.5_ concentrations are shown in Table 1. The median concentration for ZIP codes in large metropolitan counties was 13.02 μg/m^3^ [interquartile range (IQR), 9.24–17.72 μg/m^3^]. Median concentrations were 12.94 μg/m^3^ (IQR, 9.37–17.37 μg/m^3^) for ZIP codes in medium or small metropolitan counties and 12.89 μg/m^3^ (IQR, 9.31–17.29 μg/m^3^) for ZIP codes in nonmetropolitan counties.

**Table 1:** Distribution (percentiles) of estimated 24-hr average ZIP code–level PM_2.5_ concentrations (μg/m^3^) in Georgia, 1 January 2002–30 June 2010, overall and for three categories of county-level urbanicity.*^a^*

Location	1%	25%	Median	75%	99%
State of Georgia	3.45	9.31	12.94	17.43	37.35
Large metropolitan counties	3.70	9.24	13.02	17.72	36.45
Medium or small metropolitan counties	3.60	9.37	12.94	17.37	37.03
Nonmetropolitan counties	3.22	9.31	12.89	17.29	38.06
^***a***^ZIP code urbanicity classifications based on Ingram and Franco (2012) county-level population designations: “large metropolitan” (metropolitan counties with > 1 million residents) (*n *= 207 ZIP codes), “medium or small metropolitan” (metropolitan counties with 250,000–999,999 residents or < 250,000 residents, respectively) (*n *= 175 ZIP codes), and “nonmetropolitan” (counties not in a metropolitan statistical area) (*n *= 309 ZIP codes).					

**Figure 2:**
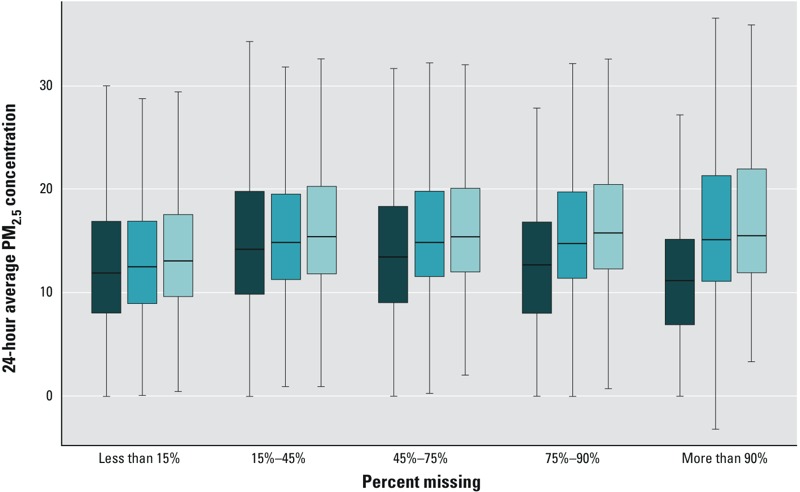
Box plots displaying daily PM_2.5_ measurements from monitoring stations (dark blue), model-estimated daily mean concentrations in ZIP codes that contain a monitor (medium blue), and model-estimated daily concentrations in the 1-km grid cells that contain a monitor (light blue). Box plots are grouped along the *x*-axis according to the proportion of model-estimated grid-level PM_2.5_ concentrations that are missing within a ZIP code on a given day. Boxes display the interquartile range of the data, with the median indicated by the dark line within each box. The whiskers extend to the most extreme point that is within 1.5 times the interquartile range of the box.

Cloud cover and errors in AOD retrieval caused grid cells to be missing data frequently. During the study period there were 177,731,759 missing out of 332,831,759 possible 1-km grid estimates (53.4% missing). Figure 2 shows a comparison of PM_2.5_ measurements from monitoring stations, the average concentrations estimated in ZIP codes where there are monitors, and the estimates from the single 1-km grid cells that overlap the monitor locations. When AOD measurements were available for most of the 1-km grid cells within a ZIP code boundary, then the measurements from the monitoring station, the average ZIP code concentration, and the concentration estimated at the grid cell that contained the monitor were similar (Figure 2). However, when a large proportion of the 1-km grid cells were missing (e.g., > 90% were missing), then the agreement between the measurements at the monitor and the AOD-based estimates differed more substantially, and the model overestimated PM_2.5_ concentrations by 4.3 μg/m^3^ on average (Figure 2).

**Figure 3:**
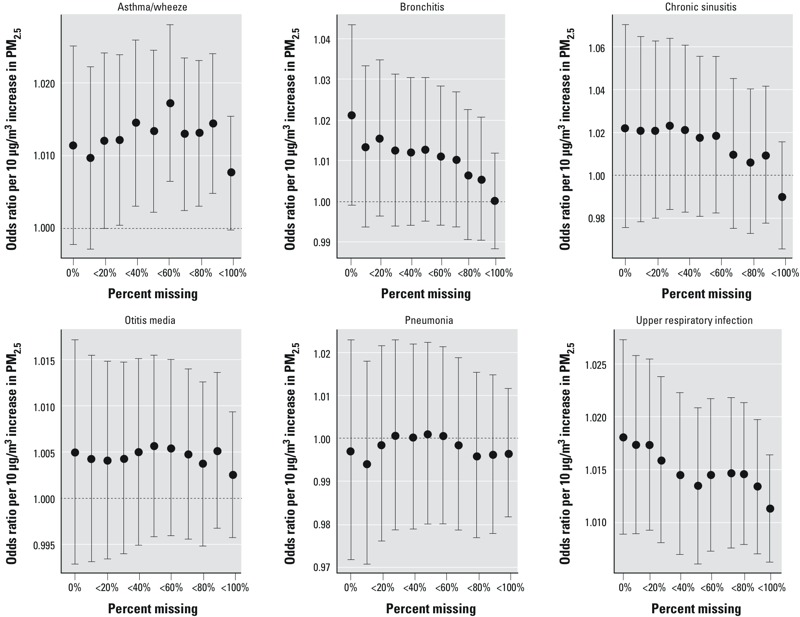
Sensitivity of odds ratios per 10-μg/m^3^ increase in same-day PM_2.5_ concentrations and ED visits for six pediatric health outcomes in Georgia, 1 January 2002–30 June 2010, according to the proportion of model-estimated grid-level PM_2.5_ concentrations that are missing within a ZIP code on each day. A percent missing (*x*-axis) of 0% means the analysis was restricted to ZIP codes that had zero missing 1-km PM_2.5_ estimates on a given day. A percent missing of < 50% means the analysis included ZIP codes that had between 0% and 50% missing 1-km PM_2.5_ estimates on a given day. A percent missing of < 100% means the analysis included all ZIP codes that had at least one non-missing 1-km PM_2.5_ estimate on a given day.

The sensitivity of the epidemiologic associations to different criteria about the proportion of missing 1-km PM_2.5_ estimates within a ZIP code is shown in Figure 3. Although the pattern between the magnitude of the OR and the proportion of missing AOD-based PM_2.5_ estimates varied by health end point, OR estimates (per 10 μg/m^3^) obtained using the least stringent missing data criterion (i.e., analyzing all ZIP codes that had at least one 1-km grid PM_2.5_ estimate) tended to be less than or equal to the OR estimates obtained using the most stringent missing data criterion (i.e., analyzing only those ZIP codes that had no missing 1-km grid PM_2.5_ estimates). These differences in the estimated associations, combined with the evidence that PM_2.5_ concentrations were overestimated when a large proportion of the grid cells were missing (Figure 2), led to the decision to only use ZIP codes when at least 30% of the 1-km PM_2.5_ estimates were available. Although a different cutoff could have been selected, we felt the 30% criterion balanced the tradeoff between sample size (i.e., wanting to use as much data as possible) and validity (i.e., avoiding the use of biased PM_2.5_ estimates). Based on this criterion, there were 1,110,827 daily ZIP code PM_2.5_ estimates included in the statistical analysis, of 2,271,317 possible (48.9%).

The number of pediatric ED visits for ZIP codes where at least 30% of the 1-km estimates were available is shown in Table 2. ED visits for asthma or wheeze were the most concentrated in the large metropolitan counties (60% in urban counties vs. 17% in nonmetropolitan counties), whereas bronchitis ED visits were proportionally more frequent in nonmetropolitan counties (30% in large metropolitan counties vs. 39% in nonmetropolitan counties).

**Table 2:** Number of pediatric emergency department visits in Georgia, 1 January 2002–30 June 2010,*^a^* stratified by county-level urbanicity.*^b^*

Outcome	Large metropolitan [*n* (%)]	Medium/small metropolitan [*n* (%)]	Nonmetropolitan [*n* (%)]	Total (*n*)
Asthma or wheeze	114,739 (60)	43,065 (23)	32,012 (17)	189,816
Bronchitis	22,535 (30)	23,419 (31)	30,289 (39)	76,243
Chronic sinusitis	6,523 (41)	3,687 (23)	5,535 (36)	15,745
Otitis media	125,474 (53)	54,095 (23)	58,264 (24)	237,833
Pneumonia	28,373 (54)	13,806 (26)	10,767 (20)	52,946
Upper respiratory infection	198,391 (48)	97,795 (24)	118,370 (28)	414,556
^***a***^ED visit counts for ZIP codes on days that have ≥ 30% nonmissing 1-km PM_2.5_ estimates. ^***b***^ZIP code urbanicity classifications based on Ingram and Franco (2012) county-level population designations: “large metropolitan” (metropolitan counties with > 1 million residents) (*n *= 207 ZIP codes), “medium or small metropolitan” (metropolitan counties with 250,000–999,999 residents or < 250,000 residents, respectively) (*n *= 175 ZIP codes), and “nonmetropolitan” (counties not in a metropolitan statistical area) (*n *= 309 ZIP codes).				

Associations between lag 0 and lag 1 PM_2.5_ concentrations and six outcome groups of pediatric ED visits are shown in Table 3. For a 10-μg/m^3^ increase in lag 0 PM_2.5_ concentrations we observed positive associations with asthma or wheeze [OR = 1.013; 95% confidence interval (CI): 1.003, 1.023] and with upper respiratory tract infections (OR = 1.015; 95% CI: 1.008, 1.022). Associations for 10-μg/m^3^ increases in lag 1 PM_2.5_ concentrations with asthma or wheeze (OR = 1.010; 95% CI: 1.000, 1.021) and upper respiratory tract infections (OR = 1.011; 95% CI: 1.004, 1.018) were slightly lower. The association between PM_2.5_ concentrations and bronchitis (lag 0 OR = 1.010; 95% CI: 0.994, 1.027) was similar in magnitude to the ORs for asthma or wheeze and upper respiratory tract infections (Table 3).

**Table 3:** Odds ratios per 10-μg/m^3^ increase in same-day PM_2.5_ concentrations and ED visits for six pediatric health outcomes in Georgia, 1 January 2002–30 June 2010 [OR (95% CI)].

Outcome group	Lag 0	Lag 1
Asthma or wheeze	1.013 (1.003, 1.023)	1.010 (1.000, 1.021)
Bronchitis	1.010 (0.994, 1.027)	1.007 (0.990, 1.024)
Chronic sinusitis	1.010 (0.975, 1.045)	0.998 (0.963, 1.034)
Otitis media	1.005 (0.996, 1.014)	0.995 (0.985, 1.004)
Pneumonia	0.999 (0.979, 1.019)	1.001 (0.981, 1.022)
Upper respiratory infection	1.015 (1.008, 1.022)	1.011 (1.004, 1.018)
Odds ratios estimated from a conditional logistic regression model with stratification by ZIP code, year, and month and with parametric control for lag 0 mean temperature, lag 0 mean humidity, and day of year using cubic polynomials; indicators for day of week, warm season, holiday, and lag holiday; and product terms between the warm season indicator and the temperature cubic polynomial, humidity cubic polynomial, day of week indicators, holiday indicators, and lag holiday indicators. Analyses are restricted to days when a ZIP code had ≥ 30% nonmissing 1-km PM_2.5_ estimates.		

**Figure 4:**
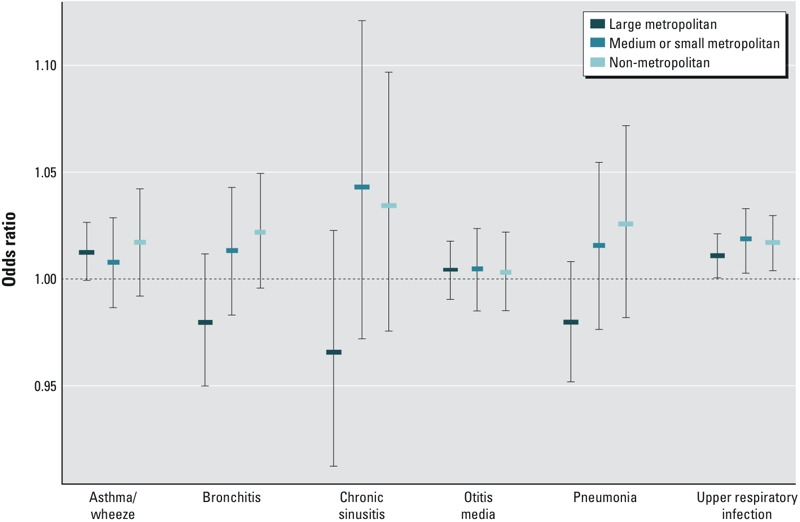
Odds ratios per 10-μg/m^3^ increase in same-day PM_2.5_ concentrations and ED visits for six pediatric health outcomes in Georgia, 1 January 2002–30 June 2010, stratified by county-level urbanicity. Odds ratios were estimated from a conditional logistic regression model with stratification by ZIP code, year, and month and with parametric control for lag 0 mean temperature, lag 0 mean humidity, and day of year using cubic polynomials; indicators for day of week, warm season, holiday, and lag holiday; and product terms between the warm season indicator and the temperature cubic polynomial, humidity cubic polynomial, day of week indicators, holiday indicators, and lag holiday indicators. Analyses were restricted to days when a ZIP code had ≥ 30% nonmissing 1-km PM_2.5_ estimates. ZIP code urbanicity classifications based on Ingram and Franco (2012) county-level population designations: “large metropolitan” (metropolitan counties with > 1 million residents), “medium or small metropolitan” (metropolitan counties with 250,000–999,999 residents or < 250,000 residents, respectively), and “nonmetropolitan” (counties not in a metropolitan statistical area).

Lag 0 associations stratified by level of urbanicity are presented in Figure 4 (for numerical results, see Table S1). These analyses did not suggest large differences in the associations of outdoor PM_2.5_ concentrations with ED visits by level of urbanicity. For the three most common outcomes (asthma/wheeze, otitis media, and upper respiratory infections) the association estimates were similar across urbanicity levels (*p*-values from the generalized Wald test for “H_0_: the three stratum-specific ORs are equal,” the null hypothesis we were testing, were 0.85, 0.99, and 0.69, respectively). In contrast, the ORs for the less common outcomes tended to be negative in urban areas and positive in less urban areas (*p*-value for differences across strata of 0.12–0.15), although estimates were imprecise. The lag 1 results similarly did not suggest large differences in associations by level of urbanicity (results not shown).

## Discussion

We observed that short-term changes in lag 0 and lag 1 PM_2.5_ concentrations were associated with ED visits for asthma or wheeze and with ED visits for upper respiratory infections. Broadly, these findings are consistent with previous literature that also shows associations between PM_2.5_ and pediatric respiratory disease (U.S. EPA 2009). We found little evidence of effect modification by level of urbanicity, even though the composition of PM_2.5_ differs in urban and rural areas (U.S. EPA 2009). For example, motor vehicle engine combustion particles comprise a larger proportion of PM_2.5_ in urban areas, whereas nonmetropolitan areas tend to have proportionately greater contributions from biogenic, forest fire, and ammonia emissions (U.S. EPA 2009). A limitation of our study is that particle composition was not characterized. Furthermore, although sample size for these two outcomes was large, we might not have detected effect modification if it was of small magnitude.

We did not observe statistically significant associations between short-term PM_2.5_ exposure and the other outcomes examined. Otitis media is a common pediatric disease that has been associated with environmental tobacco smoke and indoor wood burning (da Costa et al. 2004; DiFranza et al. 2004). A small number of studies have been conducted to investigate associations between outdoor air pollutants and otitis media; the study most similar to ours—a case-crossover analysis of 14,527 ED visits for otitis media in Edmonton, Canada—found associations for carbon monoxide and nitrogen dioxide but not for PM_2.5_ (Zemek et al. 2010). Our study, which had the advantage of having many more ED visits for otitis media (*n* = 237,833 visits) but which was exclusively focused on PM_2.5_, similarly found little evidence of this association. Results of studies on the association between chronic PM_2.5_ exposure during early life and otitis media have been inconsistent (MacIntyre et al. 2011, 2014). Associations of short-term changes in PM_2.5_ concentration with pediatric pneumonia and bronchitis, two other outcomes that we investigated in our study, have been previously reported (Barnett et al. 2005; Ostro et al. 2009), although the associations in our study were not statistically significant.

The model we used to estimate PM_2.5_ concentrations offers several advantages for epidemiologic studies of the health effects of short-term PM_2.5_ exposures. Satellite AOD measurements enable estimation of PM_2.5_ concentrations in areas where monitors are sparse or nonexistent, and using these measurements can improve estimation of day-to-day changes in PM_2.5_. Whereas other air quality models that span large spatial domains, such as CMAQ (Community Multiscale Air Quality Modeling System; https://www.epa.gov/air-research/community-multi-scale-air-quality-cmaq-modeling-system-air-quality-management) or land use regression, provide accurate characterizations of average concentrations over longer time spans, these models are less well suited for capturing the day-to-day variability that drives studies of short-term PM_2.5_ health effects (Bravo et al. 2012), although innovative methods for fusing CMAQ estimates with measurements from stationary monitors have recently been developed to help address this issue (Berrocal et al. 2012). The fine spatial scale of the MAIAC satellite data enables estimation of PM_2.5_ at 1-km grids, which enables identification of local pollution gradients, such as freeways, that are poorly characterized at coarser resolutions.

A major limitation of air quality models that incorporate satellite data, however, is the large number of missing data. In our study, 51% of daily ZIP code–level health data were not analyzed due to missing PM_2.5_ concentrations. The large number of missing data made investigation of a moving average of PM_2.5_ concentrations difficult, and as such we only examined single-day lags in our analysis. This reduction in sample size results in larger confidence intervals, although whether these missing data might also cause systematic error is presently unknown. Given that the predominant cause for missing satellite aerosol optical depth measurements in Georgia is cloud cover (Yu et al. 2015), the associations we report in this study are therefore based mostly on data from clear days. If the association between PM_2.5_ and asthma ED visits on cloudy days differs from that on clear days (i.e., effect modification by cloudiness), then the associations we report in our study should more closely approximate the clear day associations. Methods have been developed to estimate grid-level concentrations when AOD data are missing (Kloog et al. 2012c; Lee et al. 2012), although these approaches may be better suited for the investigation of health effects over longer averaging periods, as they rely heavily on land use and other parameters that do not have the same short-term variability as daily PM_2.5_ concentrations.

Some limitations of our study, which are also shared with other studies of this type, are misclassification of outcomes and error in the assignment of ambient air pollutant concentrations. We examined six different pediatric conditions, and there is likely misclassification of certain outcomes because the divisions between conditions are not always clear. For example, upper respiratory infections are a common trigger of asthma symptoms in asthmatic children, and coding practices regarding which disease is the primary cause can vary across doctors and hospitals (Strickland et al. 2010). Further, ED visits capture only a portion of the morbidity due to these conditions, and ED utilization can vary across population subgroups (Riera and Walker 2010). Although we did not observe statistically significant differences in associations with PM_2.5_ by level of urbanicity, differential ED utilization is one reason why differences by urbanicity might have been present.

There was also misclassification in the assignment of ambient air pollutant concentrations, which were linked to individual ED records based on residential ZIP code. Children may spend significant time at schools and child care facilities, which are often (but not always) located in nearby neighborhoods. This daytime mobility might result in large measurement errors for PM_2.5_ from primary sources (e.g., motor vehicle emissions), but likely has a smaller effect for secondary PM_2.5_ (e.g., sulfate particles), which are more homogenous in space (Goldman et al. 2010). Because total PM_2.5_ is a mix of primary and secondary particles, the impact of intra-day mobility in our study may be less than it would be for a study focused on traffic pollution. Furthermore, because our study is a case-crossover study with control for day-of-week, it is likely that the extent of the measurement error is similar on both case days and referent days, which would result in nondifferential error with respect to the outcome, and which would be expected to bias results toward the null.

We did not investigate confounding by other pollutants, which is a limitation of our study. Because associations between pediatric ED visits and several other pollutants have been reported in the literature, associations with PM_2.5_ might reflect an effect of another pollutant that is correlated with PM_2.5_. Adjustment for confounding by co-pollutants, as well as investigation of multipollutant joint effects, will pose challenges in settings where different air quality models are used to estimate different pollutant species, and further work in this area is needed.

## Conclusions

We observed associations between daily PM_2.5_ concentrations and pediatric ED visits for asthma or wheeze and for upper respiratory infections. The OR estimate for bronchitis was similar in magnitude to the ORs for asthma or wheeze and upper respiratory tract infections, although the confidence interval included the null. We saw little evidence for the other outcomes examined. Differences in the association estimates across levels of urbanicity were not statistically significant.

## Supplemental Material

(112 KB) PDF
