# Supplementary material for: Pediatric Emergency Visits and Short-Term Changes in PM2.5 Concentrations in the U.S. State of Georgia
Source: Environ Health Perspect. 2015 Oct 9;124(5):690–6. doi: 10.1289/ehp.1509856 (PMC4858390; doi:10.1289/ehp.1509856)
Supplement: (112 KB) PDF [file ehp.1509856.s001.acco.pdf]

Environ Health Perspect
DOI: 10.1289/ehp.1509856

Note to readers with disabilities: *EHP* strives to ensure that all journal content is accessible to all readers. However, some figures and Supplemental Material published in *EHP* articles may not conform to 508 standards due to the complexity of the information being presented. If you need assistance accessing journal content, please contact ehp508@niehs.nih.gov. Our staff will work with you to assess and meet your accessibility needs within 3 working days.

Supplemental Material

Pediatric Emergency Visits and Short-Term Changes in $PM_{2.5}$ Concentrations in the U.S. State of Georgia

Matthew J. Strickland, Hua Hao, Xuefei Hu, Howard H. Chang, Lyndsey A. Darrow, and Yang Liu

Table of Contents

Table S1. Odds ratios (OR) and 95% confidence intervals (CI) for a 10 μg/m^3 increase in same-day $PM_{2.5}$ concentrations and ED visits for six pediatric health outcomes in Georgia, 1 January 2002 – 30 June 2010, stratified by county-level urbanicity. Results presented in this Table are shown in Figure 4 of the main article.

Table S1. Odds ratios (OR) and 95% confidence intervals (CI) for a 10 μg/m^3 increase in same-day $PM_{2.5}$ concentrations and ED visits for six pediatric health outcomes in Georgia, 1 January 2002 – 30 June 2010, stratified by county-level urbanicity.[a] Results presented in this Table are shown in Figure 4 of the main article.

Outcome group	Urbanicity classification	OR (95% CI)
Asthma or wheeze	Large metropolitan	1.013 (0.999, 1.026)
	Medium/Small metropolitan	1.008 (0.986, 1.029)
	Nonmetropolitan	1.017 (0.992, 1.042)
Bronchitis	Large metropolitan	0.980 (0.950, 1.012)
	Medium/Small metropolitan	1.013 (0.984, 1.043)
	Nonmetropolitan	1.022 (0.996, 1.049)
Otitis media	Large metropolitan	1.004 (0.991, 1.017)
	Medium/Small metropolitan	1.005 (0.986, 1.024)
	Nonmetropolitan	1.003 (0.985, 1.022)
Pneumonia	Large metropolitan	0.980 (0.952, 1.008)
	Medium/Small metropolitan	1.016 (0.977, 1.056)
	Nonmetropolitan	1.026 (0.982, 1.072)
Sinusitis	Large metropolitan	0.966 (0.913, 1.022)
	Medium/Small metropolitan	1.044 (0.972, 1.121)
	Nonmetropolitan	1.034 (0.976, 1.097)
Upper respiratory infection	Large metropolitan	1.011 (1.000, 1.021)
	Medium/Small metropolitan	1.019 (1.004, 1.033)
	Nonmetropolitan	1.017 (1.004, 1.030)

[a]Odds ratios estimated from a conditional logistic regression model with stratification by ZIP code, year, and month and with parametric control for lag 0 mean temperature, lag 0 mean humidity, and day of year using cubic polynomials; indicators for day of week, warm season, holiday, and lag holiday; and product terms between the warm season indicator and the temperature cubic polynomial, humidity cubic polynomial, day of week indicators, holiday indicators, and lag holiday indicators. Analyses are restricted to days when a ZIP code had ≥30% non-missing 1-km $PM_{2.5}$ estimate.
